# Clinical significance of 206 station lymph node in transverse colon cancer

**DOI:** 10.1002/cam4.4626

**Published:** 2022-04-18

**Authors:** Yu Xin Xu, Ying Huang, Xiao Jie Wang, Dao Xiong Ye, Pan Chi

**Affiliations:** ^1^ Department of Colorectal Surgery, Union Hospital Fujian Medical University Fuzhou People's Republic of China

**Keywords:** lymph node (LN), propensity score weighting (PSW), transverse colon cancer

## Abstract

**Background:**

Lymph node (LN) metastasis is crucial in determining the prognosis and treatment options for colon cancer patients. Our work was to study whether the lymph nodes beyond D3 station in transverse colon cancer, especially 206 LN, should be dissected.

**Methods:**

A total of 225 patients within our department were reviewed. The primary and secondary endpoints were overall survival (OS) and disease‐free survival (DFS). We employed Propensity score weighting (PSW) for weighing participants to balance observed confounders between the 206^D+^ group and the 206^D−^ group.

**Results:**

The rate of metastasis in station 206 was 9.3%. Only T stage (OR, 3.009; 95% CI, 1.018–8.892), N stage (OR, 9.818; 95% CI, 1.158–83.227), and M stage (OR, 26.126; 95% CI, 1.274–535.945) were an independent risk factor for 206 station metastasis in multivariate logistic analysis. The 206^D+^ group had a similarly survival than the 206^D−^ group (3‐year DFS, 89.6% v 85.9%; *p* = 0.389; 3‐year OS, 94.6% v 85.3% *p* = 0.989). PSW further verified it. Metastasis of 206 station LN is not an independent prognostic factor, but a predictive factor of DFS.

**Conclusion:**

Station 206 LN positive is a predictive factor for DFS. Only the patient with T1‐3, N+ who is at a high risk of 206 station LN metastases should consider dissecting 206 station LN.

## Graphical abstract

Two outcomes of the analysis are (a) station 206 LN positive is a predictive factor for DFS. (b) dissection of the 206 LN station seems to have a similar OS and DFS as compared with those who did not undergo this dissection. There might be due to adjuvant chemotherapy. Only the patient with T1‐3, N+ who is a high risk of 206 station LN metastases consider dissecting 206 station LN. The topic we cover is of interest and relevance to the clinical decision of 206 LNs dissection.

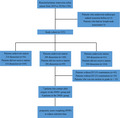

## 1 INTRODUCTION

Lymph node (LN) metastasis is a common and the main pathway for metastasis in colon cancer and is crucial in determining the prognosis and treatment options for colon cancer patients. As such, the precise range of LN dissection is necessary for surgical treatment. The primary treatment option for resectable colon cancer is colectomy involving systemic D3 station LN dissection.^1^ However, whether the lymph nodes beyond D3 station, such as 206 station LN, 204 station LN, and 214 station LN, should be exposed and the extent of their excision remains controversial.^2^, ^3^ The decision of the surgical range depends partly on the experience of the surgeon. However, station 206 LN dissection for colon cancer may cause more complications. Current researches show the 206 LN’s metastasis rate range from 5% to 13%.^4^, ^5^ According to the Japanese Classification of Colorectal Carcinoma, 206 LN is subpyloric lymph node that involves the lymph between the range from the root of the right gastroepiploic artery to the first branch and the range from anterior superior pancreaticoduodenal vein to the right gastroepiploic vein. Since the low incidence of transverse colon cancer and 206 lymph node metastases, superior 206 station LN metastasis of transverse colon cancer is rarely investigated. The American Joint Committee on Cancer (AJCC) guideline and European Society for Medical Oncology (ESMO) guideline^6^, ^7^ did not suggest whether we should dissect 206 station LN in patients with transverse colon cancer. To the best of our knowledge, few studies have compared the short‐ and long‐term outcomes of 206 station dissections so far, and the reason could be because of increased complications of 206 station dissections, low incidence of transverse colon cancer, and rare 206 station LN metastases of transverse colon cancer. Recent studies have focused more on the dissection of D2 or D3 station of LN.^1^ Therefore, we aimed to determine the clinical significance of 206 station LN dissection.

## 2 MATERIAL AND METHODS

### 2.1 Patients

We retrospectively examined the data of 230 patients who underwent transverse colectomy in our hospital between January 2010 and June 2019. Transverse colon cancer was defined as colon cancer between splenic flexure and hepatic flexure. We determine colon cancer by preoperative endoscopic pathology and postoperative surgical pathology and transverse colon cancer by surgical exploration.

**FIGURE 1:**
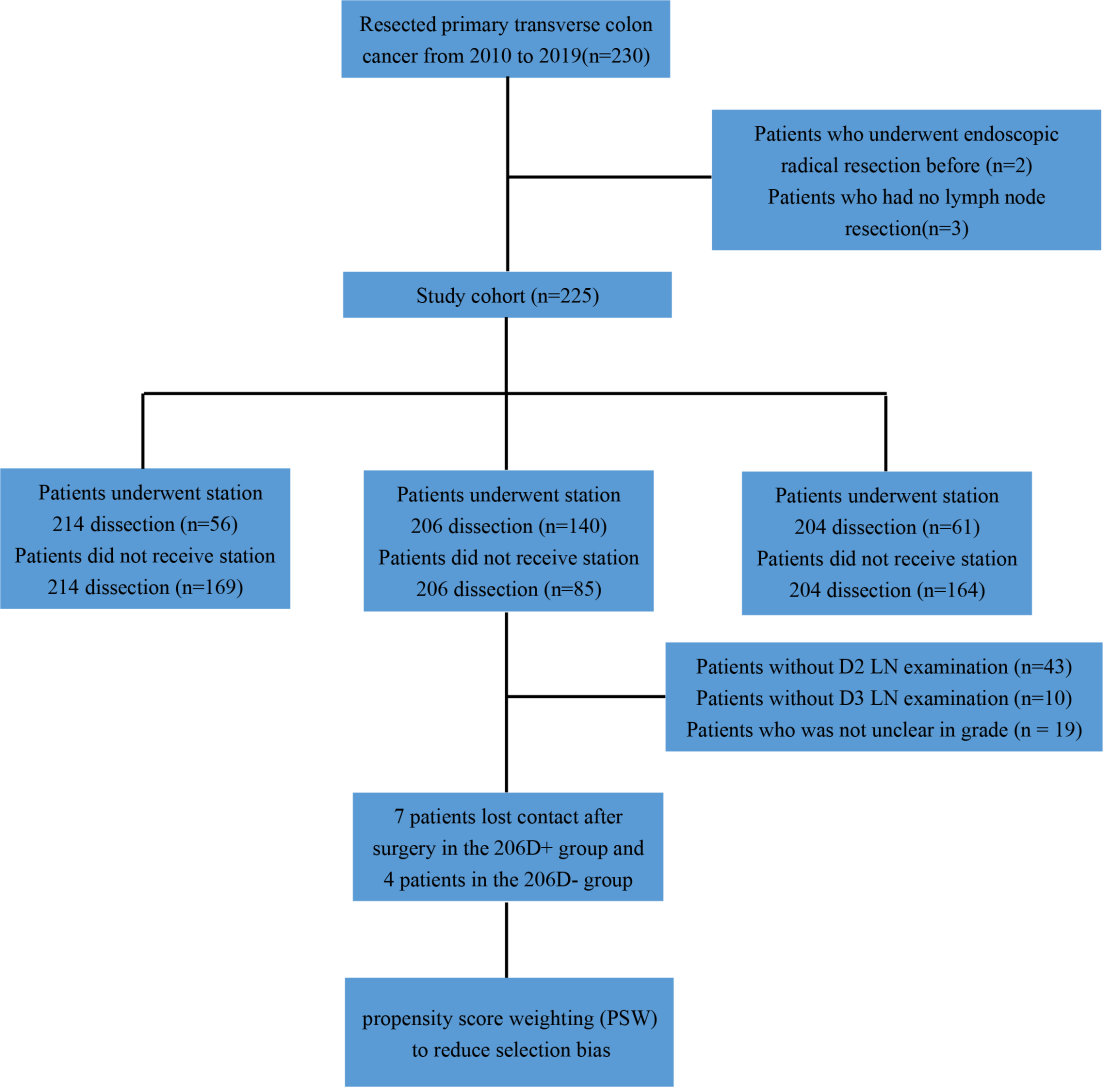
Flowchart of patient selection for this study

We included patients who gave informed consent for surgery and underwent transverse colectomy by complete mesocolic excision (CME) and did not undergo emergency surgery. Exclusion criteria were as follows: patients who underwent endoscopic radical resection before and patients who did not undergo lymph node resection. In total, we enrolled 225 patients in this study (Figure 1). Resected transverse colon cancer samples and LN were assessed by two skilled pathologists via histopathology. The LN stations were classified based on the General Rules for Clinical and Pathological Studies on Cancer of the Colon, Rectum, and Anus, 7^th^ edition, and the Japanese Classification of the Colon and Rectum (JSCCR) guidelines 2019.^8^, ^9^ Patients without D2 and D3 station LN examination and who exhibited unclear grades were removed. Tumor staging was assessed based on the seventh edition of the AJCC classification system.^10^ Histologic subtypes of adenocarcinoma were classified according to the International Classification of Diseases for Oncology Third Edition (ICD‐O‐3).

### 2.2 Follow‐ups

We collected follow‐up data from the patients and their relatives through telephone calls or via the Medical Insurance Network or from the hospital records. All the patients who were hospitalized had complete medical records. However, we lost contact with seven patients in the group that underwent 206 station LN dissection (206^D+^) and four patients in the group that did not undergo dissection (206^D−^). The 11 patients without follow‐up data were compared with the 142 patients with complete follow‐up data based on the relevant covariances. According to the results, no significant difference was observed between the two groups. Routine examinations, such as CEA and CA19‐9, chest x‐ray/computed tomography (CT) scan of the lung, CT scan/ magnetic resonance imaging (MRI) of the abdomen, and colonoscopy, were conducted after every 3 months in the first 2 years following surgery and every 6 months thereafter for 5 years. After 5 years, patients were evaluated yearly. The primary endpoint was overall survival (OS), and disease‐free survival (DFS) served as the secondary endpoint. Both OS and DFS were calculated in months.

### 2.3 Propensity score weighting

We employed the propensity score weighting (PSW) to balance the observed confounders between the 206^D−^ group and 206^D+^ group. The participants were weighed based on their estimated probability of exposure given confounders (the propensity score).^11^, ^12^, ^13^ Given that every individual has a different weight, if the weighting coefficient is 1.6, it will be considered as 1.6 people. The PSW is used to construct a virtual standard population by incorporating individual weights. The balance of the baseline characteristics was evaluated by standardized mean differences (SMD). This usually leads to variations between the number of valid cases and the total count in the cross‐tabulation table because of rounding off of the cell counts (Table 2). However, the variations are usually minimal. Besides, the overall percentage remains 100% of the patients in the propensity score weighting (PSW) analysis.^14^ Also, the 11 patients without follow‐up data were excluded from inverse probability of treatment weighting (IPTW) and standardization mortality weighting (SMRW) analysis. Propensity scores for all patients were determined using multiple logistic regression^15^, ^16^ and the covariates were as follows: sex, age, pathological N (pN) stage, pathological T (pT) stage, pathological M (pM) stage, pathological tumor‐node‐metastasis (pTNM) stage, histology, grade, and LN total subgroup. Comparing with traditional propensity score matching, IPTW and SMRW could use the data more effectively.

### 2.4 Data analysis

Data analyses were conducted using R 3.6.1. A Chi‐square test was employed to compare categorical variables, whereas t‐test was applied to compare continuous variables. The association between station 206 metastasis and risk factors was evaluated by a multivariate analysis performed using a logistic regression model. Estimation of survival was done using the Kaplan‐Meier method followed by the log‐rank test. The hazard ratio (HR) and 95% CI were determined using Multivariate Cox proportional hazards regression analysis. Statistical significance was set at *p* < 0.05.

## 3 RESULTS

### 3.1 Distribution of LN involvement and hierarchical distribution of LN involvement based on T stage

**FIGURE 2:**
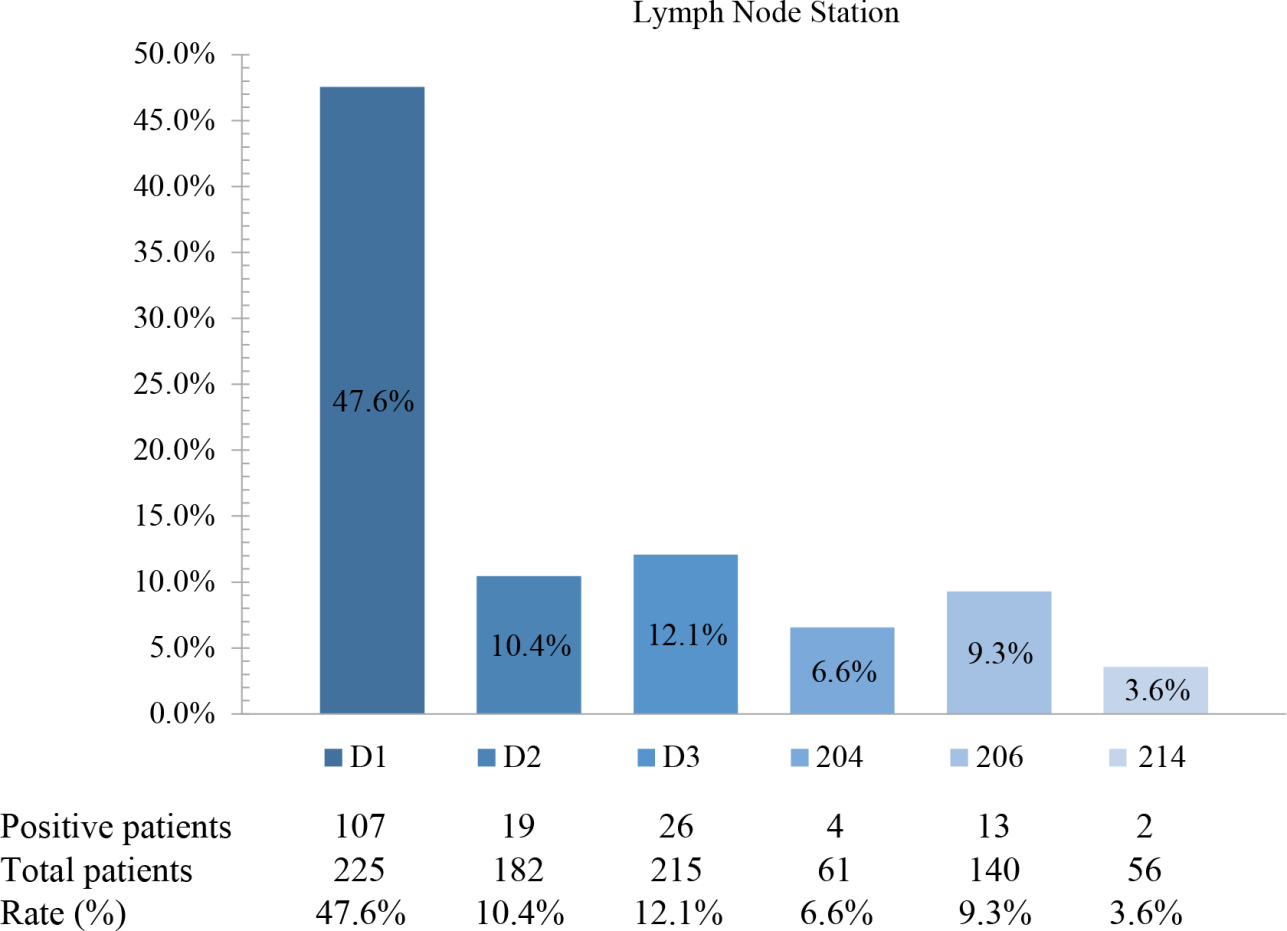
Metastasis rate of each lymph node station (D1, D2, D3, 204, 206, 214)

Among 225 patients all had D1, D2, and D3 station dissection, but not all had lymph node examination for D2 and D3 station. Besides, 140 and 56 patients had 206 and 214 station dissection among 225 patients. Moreover, 61 patients had 204 station examination. Of the 140 patients with 206 station LN dissection, 13 (9.3%) had 206 station involvement. Of the 56 patients with 214 station LN dissection, 2 (3.6%) had 214 station involvement. Because we did not have a routine inspection of 204 station LN, there are only 61 patients with 204 station LN examination and 4 (6.6%) had 204 station involvement (Figure 2). Tables S1 and S2 summarize the frequency of node involvement per station in transverse colon cancer using hierarchical analysis base on the T stage. Among 225 patients, all T1 stage patients did not have D1, D2, and D3 station LN involvement. Similarly, all T1 and T2 stage patients did not have 204 and 206 station LN involvement, moreover, 214 station LN involvement only could be found among patients with T4 stage. The skip metastasis rate Is shown in the Table S3. In this study, 120 cases were negative for D1 station lymph nodes, for which 3.3% were positive for D2 station nodes. 165 cases were negative for D2 station lymph nodes, for which 9.7% were positive for D3 station nodes.

### 3.2 Analysis of risk factor for 206 station lymphatic metastasis

The 206 station LN metastasis was significantly correlated with pT, pN, pM, and AJCC7th TNM stage (pT stage, *p* < 0.001; pN stage, *p* = 0.003; pM stage, *p* = 0.035; pTNM stage, *p* = 0.005). Considering collinearity, the factors that were statistically significant besides pTNM stage were analyzed further using multivariate logistic analysis. Based on the results, T stage (odds ratio, 3.009; 95% CI, 1.018–8.892; *p* = 0.046), N stage (odds ratio, 9.818; 95% CI, 1.158–83.227; *p* = 0.036), and M stage (odds ratio, 26.126; 95% CI, 1.274–535.945; *p* = 0.034) were independently associated with 206 station LN metastasis (Table 1).

**TABLE 1:** Univariate and multivariate analysis of correlation between clinicopathological factors and 206 station lymph node metastasis

Characteristic	No.	206 station lymph node metastasis no. (%)		Univariate analysis	Multivariate analysis		
		Positive	Negative	*p*	OR	95% CI	*p*
Total	103	8	95				
Sex				0.580			
Male	63	6 (75.0)	57 (60.0)				
Female	40	2 (25.0)	38 (40.0)				
Age, years				0.411			
<65	68	6 (75.0)	62 (65.3)				
≥65	35	2 (25.0)	33 (34.7)				
pT stage				0.001	3.009	(1.018–8.892)	0.046
T1	2	0 (0.0)	2 (2.1)				
T2	3	0 (0.0)	3 (3.2)				
T3	81	2 (25.0)	79 (83.2)				
T4a	6	2 (25.0)	4 (4.2)				
T4b	11	4 (50.0)	7 (7.4)				
pN stage				0.003	9.818	(1.158–83.227)	0.036
N0	44	0 (0.0)	44 (46.3)				
N1	39	2 (25.0)	37 (38.9)				
N2	20	6 (75.0)	14 (14.7)				
pM stage				0.035	26.126	(1.274–535.945)	0.034
M0	97	6 (75.0)	91 (95.8)				
M1	6	2 (25.0)	4 (4.2)				
pTNM stage				0.005			
I		0 (0.0)	4 (4.2)				
II							
IIA		0 (0.0)	37 (38.9)				
IIB		0 (0.0)	2 (1.1)				
IIC		0 (0.0)	0 (0.0)				
III							
IIIA		0 (0.0)	1 (1.1)				
IIIB		1 (12.5)	38 (40.0)				
IIIC		5 (35.7)	9 (9.5)				
IV		2 (25.0)	4 (4.2)				
Grade				0.199			
I	4	1 (12.5)	3 (3.2)				
II	97	7 (87.5)	90 (94.7)				
III	2	0 (0.0)	2 (2.1)				
LN total subgroup				0.879			
<12	1	0 (0.0)	1 (1.1)				
≥12	102	8 (100.0)	94 (98.9)				
Histology				0.757			
Tubular or papillary adenocarcinoma	78	6 (75.0)	72 (75.8)				
Adenocarcinoma	8	1 (12.5)	7 (7.4)				
Mucinous adenocarcinoma	16	0 (0.0)	16 (16.8)				
Signet ring cell carcinoma	1	1 (12.5)	0 (0.0)				
D1 metastasis				0.749			
No	52	0 (0.0)	52 (54.7)				
Yes	51	8 (100.0)	43 (45.3)				
D2 metastasis				0.238			
No	91	6 (75.0)	85 (89.5)				
Yes	12	2 (25.0)	10 (10.5)				
D3 metastasis				0.393			
No	88	6 (75.0)	82 (86.3)				
Yes	15	2 (25.0)	13 (13.7)				
Site				0.620			
Hepatic flexure	75	5 (62.5)	70 (73.7)				
Transverse colon	26	3 (37.5)	23 (24.2)				
Splenic flexure	2	0 (0.0)	2 (2.1)				

### 3.3 Baseline data before and after weighting

The baseline data of the patients are summarized in Table 2 (*n* = 142). In total, 96 (67.6%) and 46 (32.4%) patients were allocated to the 206^D+^ group and 206^D−^ group, respectively. Prior to weighting, the difference was observed in relation to the pTNM stage (*p* = 0.034) and Site (*p* < 0.001); after SMRW and IPTW, similar results were observed between the two groups (*p* > 0.05; Table 2). The standardized mean difference (SMD) of the three groups is shown in Figure S1.

**TABLE 2:** Patients' baseline data before, after propensity score weighting (PSW) and standardization mortality weighting (SMRW)

Characteristic	Entire Cohort				SMRW				IPTW			
	206D (−) Group	206D (+) Group	*p*	SMD	206D (−) Group	206D (+) Group	*p*	SMD	206D (−) Group	206D (+) Group	*p*	SMD
Total	46	96			24.7	25.4			126.9	123.9		
Sex			1.000	0.012			0.975	0.007			0.446	0.163
Male	28 (60.9)	59 (61.5)			16.5 (66.7)	17.0 (67.1)			89.3 (70.4)	77.7 (62.7)		
Female	18 (39.1)	37 (38.5)			8.2 (33.3)	8.4 (32.9)			37.6 (29.6)	46.2 (37.3)		
Age, years			0.625	0.121			0.657	0.109			0.426	0.189
<65	28 (60.9)	64 (66.7)			13.9 (56.3)	15.7 (61.7)			70.0 (55.2)	79.8 (65.7)		
≥65	18 (39.1)	32 (33.3)			10.8 (43.7)	9.7 (38.3)			56.9 (44.8)	44.1 (34.3)		
pT stage			0.087	0.494			0.994	0.060			0.956	0.123
T1	2 (4.3)	2 (2.1)			1.2 (4.8)	1.0 (4.1)			3.3 (2.6)	3.0 (2.4)		
T2	5 (10.9)	3 (3.1)			1.0 (4.0)	1.2 (4.6)			6.2 (4.7)	4.2 (3.4)		
T3	27 (58.7)	76 (79.2)			17.8 (72.1)	18.7 (73.5)			99.4 (78.3)	95.8 (77.3)		
T4a	9 (19.6)	13 (13.5)			4.7 (19.2)	4.5 (17.8)			15.3 (12.0)	18.9 (15.2)		
T4b	3 (6.5)	2 (2.1)			0 (0.0)	0 (0.0)			3.0 (2.4)	2.0 (1.6)		
pN stage			0.255	0.379			0.788	0.066			0.143	0.422
N0	29 (63.0)	45 (46.9)			15.1 (61.2)	14.7 (58.0)			64.3 (50.7)	60.4 (48.7)		
N1	13 (28.3)	33 (34.4)			9.6 (38.8)	10.7 (42.0)			58.6 (46.2)	45.5 (36.7)		
N2a	2 (4.3)	7 (7.3)			0 (0.0)	0 (0.0)			2.0 (1.6)	7.0 (5.7)		
N2b	2 (4.3)	11 (11.5)			0 (0.0)	0 (0.0)			2.0 (1.6)	11.0 (8.9)		
pM stage			0.092	0.335			0.960	0.013			0.482	0.150
M0	39 (84.8)	91 (94.8)			22.2 (89.9)	25.5 (89.5)			112.5 (88.7)	115.2 (93.0)		
M1	7 (15.2)	5 (5.2)			2.5 (10.1)	2.7 (10.5)			14.4 (11.3)	8.7 (7.0)		
pTNM stage			0.034	0.722				0.060			0.376	0.492
I	5 (10.9)	4 (4.2)			2.2 (8.8)	2.2 (8.7)			8.8 (6.3)	7.3 (5.2)		
II	21 (45.7)	39 (40.6)			12.7 (47.3)	11.3 (44.5)			55.5 (39.7)	52.7 (37.5)		
IIA	17 (37.0)	37 (38.5)			9.8 (39.7)	9.5 (37.6)			48.2 (34.5)	48.3 (34.4)		
IIB	3 (6.5)	2 (2.1)			1.9 (7.6)	1.8 (6.9)			5.3 (3.8)	4.4 (3.1)		
IIC	1 (2.2)	0 (0.0)			0 (0.0)	0 (0.0)			2.0 (1.4)	0.0 (0.0)		
III	13 (28.3)	48 (49.9)			8.4 (33.9)	9.9 (35.2)			60.7 (43.6)	70.6 (50.2)		
IIIA	0 (0.0)	1 (1.0)			0 (0.0)	0 (0.0)			0.0 (0.0)	1.0 (0.7)		
IIIB	12 (26.1)	34 (35.4)			8.4 (33.9)	9.2 (36.3)			59.6 (42.8)	55.5 (39.5)		
IIIC	1 (2.2)	13 (13.5)			0 (0.0)	0 (0.0)			1.1 (0.8)	14.1 (10.0)		
IV	7 (15.2)	5 (5.2)			2.5 (8.9)	2.7 (10.5)			14.4 (10.3)	9.8 (7.0)		
Grade			0.520	0.230			0.892	0.018			0.365	0.189
I	3 (6.5)	4 (4.2)			0.3 (1.3)	0.3 (1.1)			3.3 (2.6)	4.3 (3.5)		
II	43 (93.5)	90 (93.8)			24.4 (98.7)	25.1 (98.9)			123.6 (97.4)	117.6 (94.9)		
III	0 (0.0)	2 (2.1)			0 (0.0)	0 (0.0)			0.0 (0.0)	2.0 (1.6)		
LN total subgroup			0.510	0.205			0.984	0.004			0.678	0.062
<12	2 (4.3)	1 (1.0)			0.4 (1.7)	0.4 (4.6)			2.4 (1.9)	1.4 (1.1)		
≥12	44 (95.7)	95 (99.0)			24.3 (98.3)	25.0 (98.4)			124.5 (98.1)	122.5 (98.9)		
Histology			0.355	0.324			0.987	0.028			0.826	0.145
Tubular or papillary adenocarcinoma	30 (65.2)	73 (76.0)			17.9 (72.4)	18.2 (71.6)			91.8 (72.3)	92.0 (74.3)		
Adenocarcinoma	7 (15.2)	7 (7.3)			1.2 (4.7)	1.1 (4.4)			8.2 (6.4)	8.1 (6.5)		
Mucinous adenocarcinoma	9 (19.6)	15 (15.6)			5.7 (22.9)	6.1 (24.2)			26.9 (21.2)	22.7 (18.4)		
Signet ring cell carcinoma	0 (0.0)	1 (1.0)			0.0 (0.0)	0.0 (0.0)			0.0 (0.0)	1.0 (0.8)		
Site			<0.001	1.117			0.909	0.028			0.076	0.478
Hepatic flexure	14 (30.4)	69 (71.9)			11.9 (48.1)	11.9 (46.7)			74.2 (58.5)	92.0 (74.3)		
Transverse colon	19 (41.3)	27 (28.1)			12.8 (51.9)	13.5 (53.3)			39.7 (31.3)	42.5 (34.3)		
Splenic flexure	13 (28.3)	0 (0.0)			0.0 (0.0)	0.0 (0.0)			13.0 (10.2)	0 (0.0)		

### 3.4 Survival of patients before and after weighting

At the end of this study, total deaths and metastasis/recurrences during follow‐ups were 13 and 10, respectively. We recorded eight deaths and eight metastasis/recurrences in the 206^D+^ group. In the 206^D−^ group, there were five deaths and two metastasis/recurrence. The 3‐year DFS rates were 89.6% in the 206^D+^ group and 85.9% in the 206^D−^ group. The 3‐year OS rates in the two groups were 94.6% and 85.3%. The log‐rank test indicated that the 206^D+^ group had a similarly survival compared with the 206^D−^ group (DFS, *p* = 0.389; OS, *p* = 0.989; Figure S2A,B). Following IPTW, the DFS and OS were similar in the 206^D+^ group relative to the 206^D−^ group (*p* = 0.091 and 0.313, respectively; Figure S2C,D). After SMRW, the DFS and OS were also similar in the 206^D+^ group relative to the 206^D−^ group (*p* = 0.253 and 0.141, respectively; Figure S2E,F).

### 3.5 Survival factor analysis

Several variables, including the status of 206 station LN positive, pT stage, pN stage, pTNM stage, D1 station metastasis, and D3 station metastasis, were all significant risk factors for DFS based on the univariate analysis (*p* = 0.001, *p* < 0.001, *p* < 0.001, *p* = 0.003, and *p* = 0.041, 0.036, respectively), and status of pT stage, pN stage, pM stage, pTNM stage, and D1 station metastasis were all significant risk factors for OS (*p* < 0.001, *p* < 0.001, *p* = 0.002, *p* < 0.001,and *p* = 0.022, respectively) based on the univariate analysis (Table 3). Further multivariate analysis and predictive factors selected by Akaike information criterion (AIC) indicated that the status of 206 station LN positive was not an independent risk factor for DFS and OS, but a predictive factor for DFS (HR, 1.887; 95% CI, 1.596–2.178; *p* = 0.029), in addition to pT stage and pTNM stage (Table 3).

**TABLE 3:** Cox regression analysis of prognostic factors select by univariate and multivariate and predict factors select by AIC in transverse colon cancer

Predictor	OS						DFS					
	Univariate analysis		Multivariate analysis		Predictive factors selected by AIC		Univariate analysis		Multivariate analysis		Predictive factors selected by AIC	
	*p*	HR (95% CI)	*p*	HR (95% CI)	*p*	HR (95% CI)	*p*	HR (95% CI)	*p*	HR (95% CI)	*p*	HR (95% CI)
Sex	0.277	0.547 (0.184 to 1.627)					0.971	1.018 (0.534 to 1.502)				
Age	0.878	1.003 (0.981 to 1.025)					0.470	0.983 (0.959 to 1.007)				
pT stage	<0.001	4.599 (4.242 to 4.956)	0.002	3.553 (1.566 to 8.061)	<0.001	5.896 (5.394 to 6.399)	<0.001	4.924 (4.522 to 5.326)	0.005	2.738 (1.359 to 5.513)	0.001	2.999 (2.663 to 3.335)
pN stage	<0.001	2.657 (2.412 to 2.902)	0.007	2.795 (1.326 to 5.890)	<0.001	3.194 (2.863 to 3.524)	<0.001	2.789 (2.514 to 3.064)	0.370	1.402 (0.670 to 2.932)		
pM stage	0.002	6.265 (5.687 to 6.843)	0.027	4.313 (1.194 to 15.572)	0.002	9.818 (9.050 to 10.586)						
pTNM stage	<0.001	2.025 (1.818 to 2.232)					0.003	1.874 (1.661 to 2.087)	0.010	1.691 (1.136 to 2.519)	0.001	1.758 (1.581 to 1.935)
Grade	0.099	0.283 (0.063 to 1.269)					0.257	0.435 (0.103 to 1.832)				
Histology	0.252	1.421 (0.779 to 2.590)			0.056	1.974 (0.984 to 3.959)	0.652	1.183 (0.811 to 1.555)				
D1 metastasis	0.022	3.974 (3.371 to 4.577)	0.591	0.609 (0.100 to 3.726)			0.041	4.110 (3.419 to 4.801)	0.704	0.744 (0.161 to 3.426)		
D2 metastasis	0.184	2.778 (0.615 to 12.568)					0.090	2.927 (0.844 to 10.146)				
D3 metastasis	0.134	2.681 (0.737 to 9.751)			0.112	0.246 (0.043 to 1.427)	0.036	4.252 (3.561 to 4.943)	0.993	0.994 (0.281 to 3.518)		
Site	0.931	1.035 (0.475 to 2.257)					0.857	1.080 (0.652 to 1.508)				
Station 206 dissection	0.988	0.991 (0.421 to 1.561)					0.856	0.890 (0.244 to 1.536)				
Station 206 positive	0.176	1.974 (0.735 to 5.305)			0.135	1.857 (0.820 to 4.153)	0.001	3.315 (1.584 to 6.939)	0.089	1.704 (0.922 to 3.149)	0.029	1.887 (1.596 to 2.178)

### 3.6 Decision of 206 station lymph node

**FIGURE 3:**
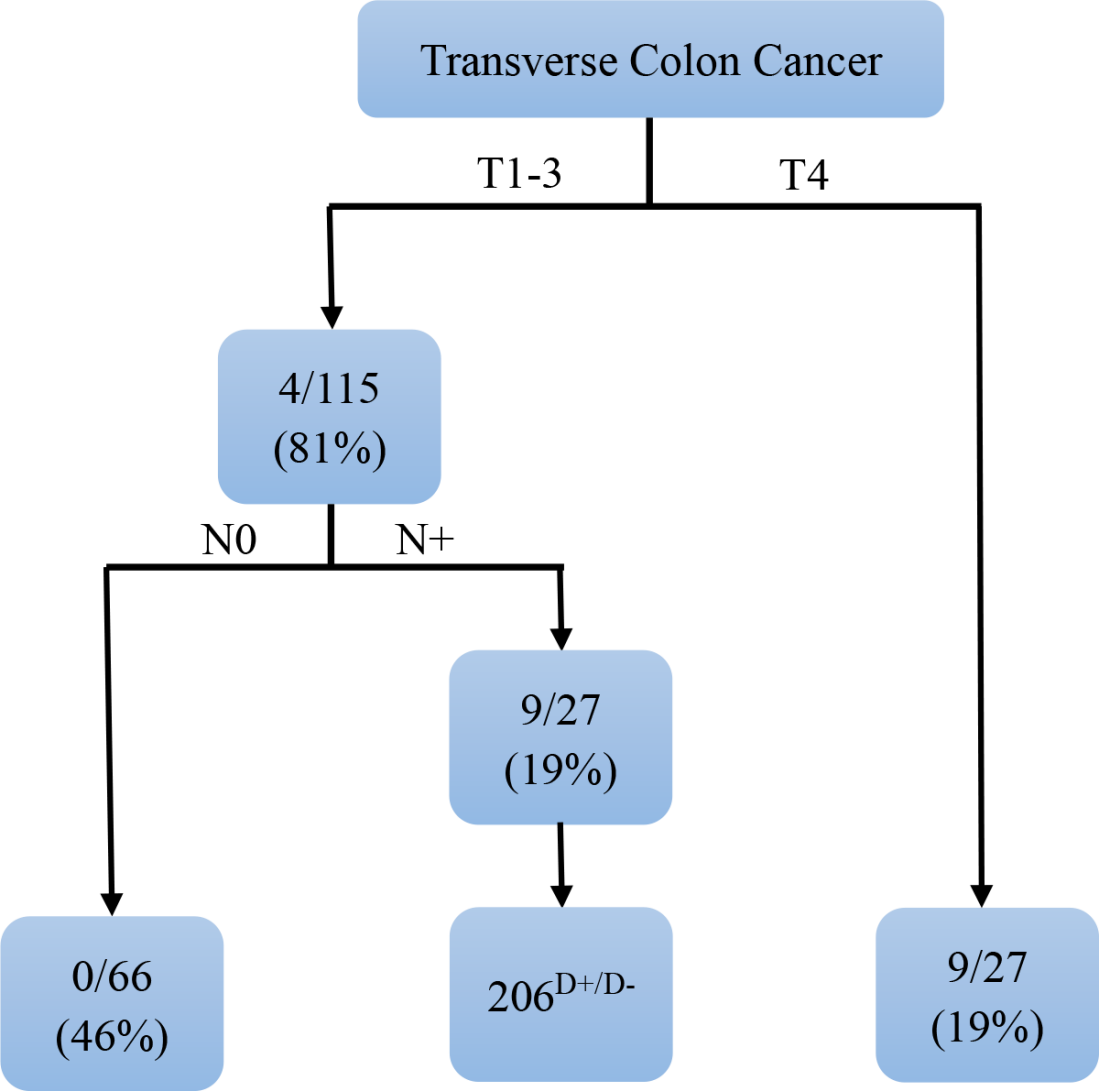
Pruned decision tree classifier based on patients’ overall survival

Based on the analysis of the decision tree, we found that only T1‐3, N+ patients may benefit from the dissection of 206 station lymph node (Figure 3). There are 13 patients who died during the follow‐up period, which were nine patients with T4 and four patients with T1‐3, N+.

## 4 DISCUSSION

Metastasis of cancer cells is one of the main determinants of poor prognosis in colon cancer.^17^, ^18^, ^19^ Given the vital role played by LN in the metastasis of colon cancer cells, complete removal of LN is crucial. Concerning transverse colon cancer, the NCCN guideline does not suggest a minimal dissection of lymph node yield.^1^, ^20^, ^21^, ^22^, ^23^ The Japanese Classification of Colorectal Carcinoma emphasized the removal of at least D3 for colon cancers with cN(+) or cN(−) and above cT2.^8^ Thereby resulting in limited data regarding the dissection of 206 station LN, as well as its effect on prognosis.^24^, ^25^ As such, whether it should be inside the omental artery remains a secret when dissecting the gastrocolic ligament. Given this, we aimed to retrospectively review the clinical significance of removing 206 station LN.

In the present study, the common sites for metastasis of LN among the 225 patients with transverse colon cancer involved 206 station LN. This observation was consistent with those reported previously.^2^, ^3^ According to univariate analysis, 206 station LN metastasis had a significant correlation with T stage, N stage, M stage, and pTNM stage (*p* < 0.05). Also, station 206 metastasis occurred most often at the higher level of the pTNM stage (*p* < 0.05). Given the collinearity, we analyzed further the statistically significant factors, other than the pTNM stage using multivariate logistic analysis. Based on multivariate analysis, only T stage (HR = 3.009, *p* < 0.05), N stage (HR = 9.818, *p* < 0.05), and M stage (HR = 26.126, *p* < 0.05) emerged as independent risk factors for 206 station LN metastasis. So patients with higher T stage, N stage, and M stage more easily have 206 station LN metastasis and more likely need to dissect 206 station LN. However, there is still a need to conduct prospective studies involving a large sample size to validate these results.

In the current study, the 206^D+^ and the 206^D−^ groups had similar DFS and OS rates. Furthermore, based on multivariate analysis and predictive factors selected by AIC, station 206 LN positive status was not an independent risk factor for DFS and OS, but a predictive factor for DFS. This could be because although 206 station LN dissection is effective in removing localized LN metastasis, undetected micrometastases, and reducing recurrence, adjuvant treatment can achieve the same effect. Herein, we applied the PSW method to avoid selection bias. The method was useful in comparing the prognostic value of resection for transverse colon cancer between the 206^D+^ group and the 206^D−^ group. After weighting, the 206^D+^ group still had a similar survival rate comparing with 206^D−^ group. Given this, 206 station LN should not be dissected routinely, which means surgery should not be inside the omental artery when dissecting the gastrocolic ligament. Only the patient with T1‐3, N+ who is a high risk of 206 station LN metastases consider being inside the omental artery when dissecting gastrocolic ligament (Figure 3). Even, patients who are at a high risk of 206 station LN metastases could be outside the omental artery when dissecting gastrocolic ligament and only have a biopsy of 206 station LN, such as patients with T4 since these patients could not benefit from the dissection of 206 station lymph node. However, it may be a risky decision since patients with positive 206 station LN have worse long‐term prognoses and we are not sure whether the dissection of 206 station LN could be replaced by adjuvant treatment. Therefore, it is still a need to conduct further studies with large sample sizes to verify these results.

The current study was limited in the following areas. First, this study was retrospective and involved a single center, which could have influenced our results despite using PSW to balance the variables. Second, patients with 206 station LN dissection positive were few, and this could have brought bias in the selection. Third, we lacked follow‐up data for few patients. Even though no significant difference was observed between the patients without follow‐up data (11) and those with complete follow‐up data (142) regarding the relevant covariances, differences that could be produced in the data were not observed between the 206^D+^ and 206^D−^ groups. The patients without follow‐up data in the 206^D−^ group exhibited relatively higher TNM stages than those in the 206^D+^ group. Therefore, there is still a need to conduct a multicenter randomized clinical trial to further confirm these findings.

## 5 CONCLUSION

Dissection of the 206 station LN has a similar prognosis in comparison to those who did not undergo this dissection. However, station 206 LN positive is a predictive factor for DFS. Only the patient with T1‐3, N+ who is at a high risk of 206 station LN metastases should consider dissecting 206 station LN.

## AUTHOR'S CONTRIBUTIONS

YuXin Xu processed the data and carried out computational simulations. XiaoJie Wang, Ying Huang, and DaoXiong Ye helped with data collections and analyses. YuXin Xu, Pan Chi, XiaoJie Wang, Ying Huang, and DaoXiong Ye analyzed the results. YuXin Xu, Pan Chi, and XiaoJie Wang drafted the manuscript.

## CONFLICT OF INTEREST

All authors have no conflict of interest to declare.

## CONSENT FOR PUBLICATION

All authors reviewed and approved the manuscript.

## ETHICS APPROVAL AND CONSENT TO PARTICIPATE

The Medical Ethics Committee of Fujian Medical University (FJMU) approved this study.

## Supporting information


Table S1

Table S2

Table S3

Figure S1

Figure S2

## Data Availability

Data sharing is not applicable to this article as no new data were created or analyzed in this study.
