# Supplementary material for: Clinical significance of 206 station lymph node in transverse colon cancer
Source: Cancer Med. 2022 Apr 18;11(12):2366–76. doi: 10.1002/cam4.4626 (PMC9189469; doi:10.1002/cam4.4626)
Supplement: Supplementary file 1 — Table S1 [file CAM4-11-2366-s005.docx]

D1, D2, and D3 metastasis percent

|  | T1 | T2 | T3 | T4 | Total |
| --- | --- | --- | --- | --- | --- |
| D1 (n=225) | 0 | 1.3% | 32.9% | 13.3% | 47.6% |
| D2 (n=182) | 0 | 0.5% | 6.6% | 3.3% | 10.4% |
| D3 (n=215) | 0 | 0.5% | 7.0% | 4.7% | 12.1% |
