# Supplementary material for: Clinical significance of 206 station lymph node in transverse colon cancer
Source: Cancer Med. 2022 Apr 18;11(12):2366–76. doi: 10.1002/cam4.4626 (PMC9189469; doi:10.1002/cam4.4626)
Supplement: Supplementary file 2 — Table S2 [file CAM4-11-2366-s003.docx]

204, 206 and 214 metastasis percent

|  | T1 | T2 | T3 | T4 | Total |
| --- | --- | --- | --- | --- | --- |
| 204 (n=61) | 0 | 0 | 4.9% | 1.6% | 6.6% |
| 206 (n=140) | 0 | 0 | 4.3% | 5.0% | 9.3% |
| 214 (n=56) | 0 | 0 | 0 | 3.6% | 3.6% |
