# Supplementary material for: Clinical significance of 206 station lymph node in transverse colon cancer
Source: Cancer Med. 2022 Apr 18;11(12):2366–76. doi: 10.1002/cam4.4626 (PMC9189469; doi:10.1002/cam4.4626)
Supplement: Supplementary file 3 — Table S3 [file CAM4-11-2366-s001.docx]

D2 and D3 skip metastasis percent

|  | T1 | T2 | T3 | T4 | Total |
| --- | --- | --- | --- | --- | --- |
| D2 (n=118) | 0 | 0 | 1.7% | 1.7% | 3.4% |
| D3 (n=163) | 0 | 0.6% | 4.3% | 4.9% | 9.8% |
