# Supplementary figures and images for: Clinical significance of 206 station lymph node in transverse colon cancer
Source: Cancer Med. 2022 Apr 18;11(12):2366–76. doi: 10.1002/cam4.4626 (PMC9189469; doi:10.1002/cam4.4626)

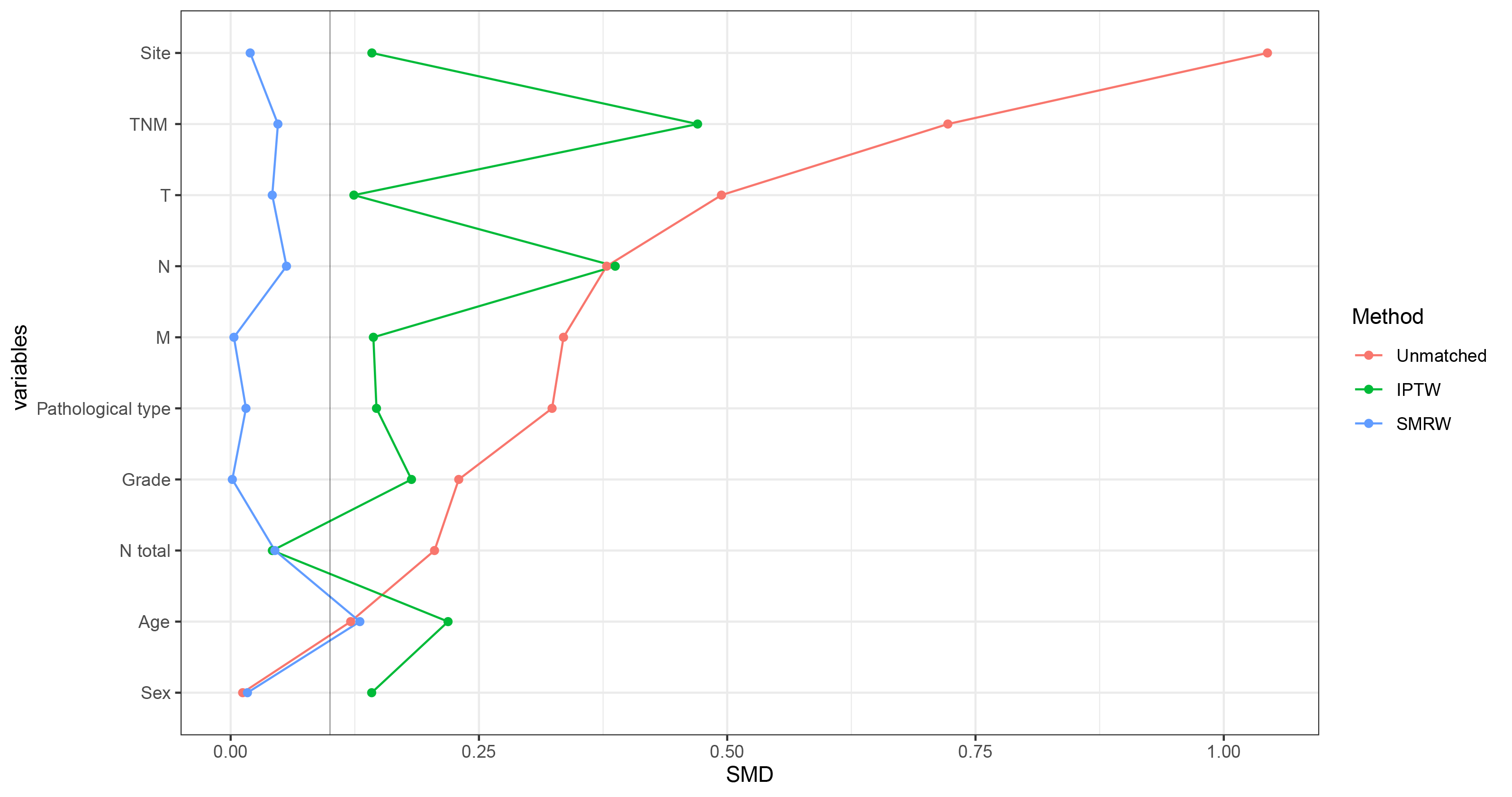

Supplement: Supplementary file 4 — Figure S1 [file CAM4-11-2366-s004.tif]

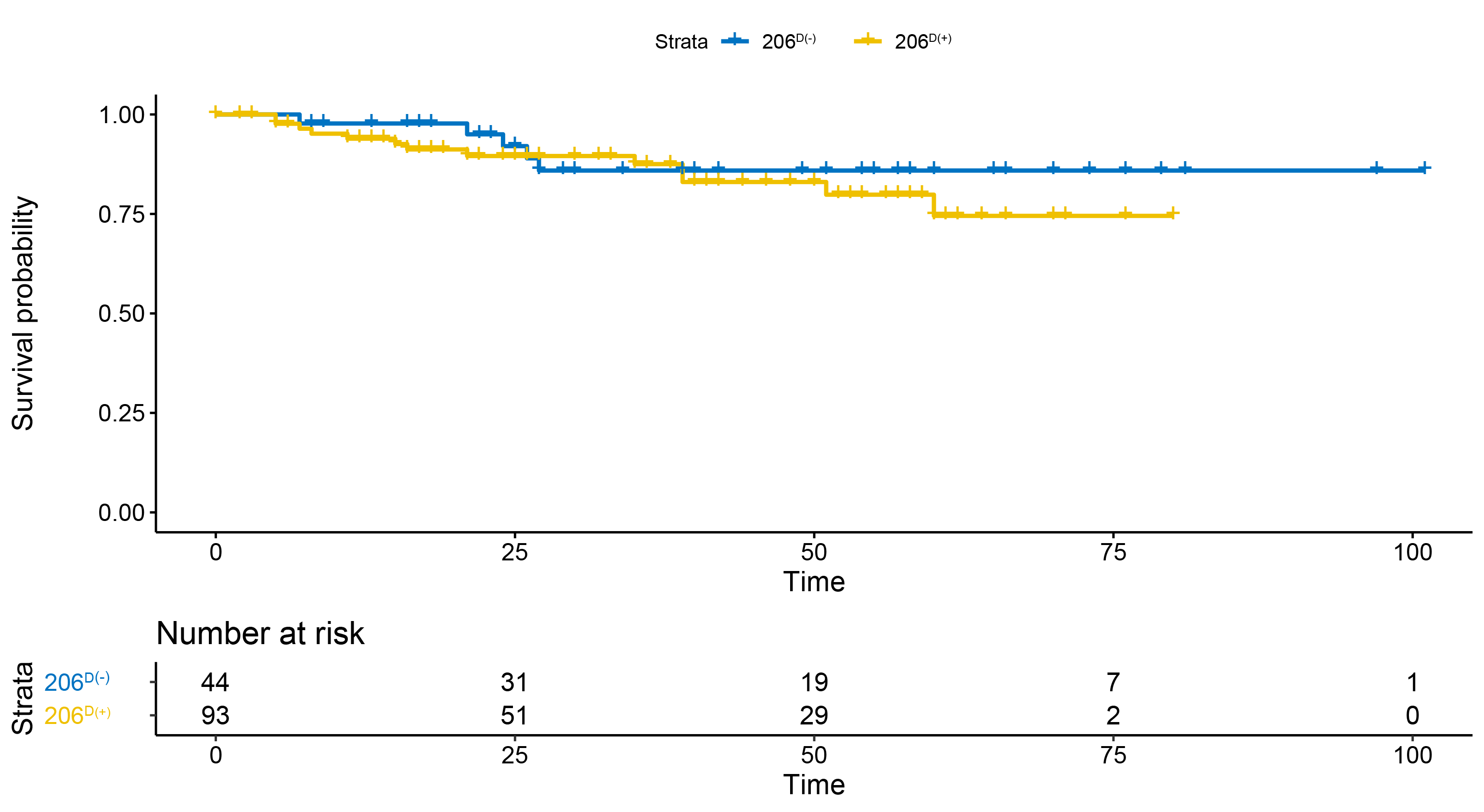

Supplement: Supplementary file 5 — Figure S2 [file CAM4-11-2366-s002.zip › figure/CAM4_4626_Supplementary_Figure2_a.tif]

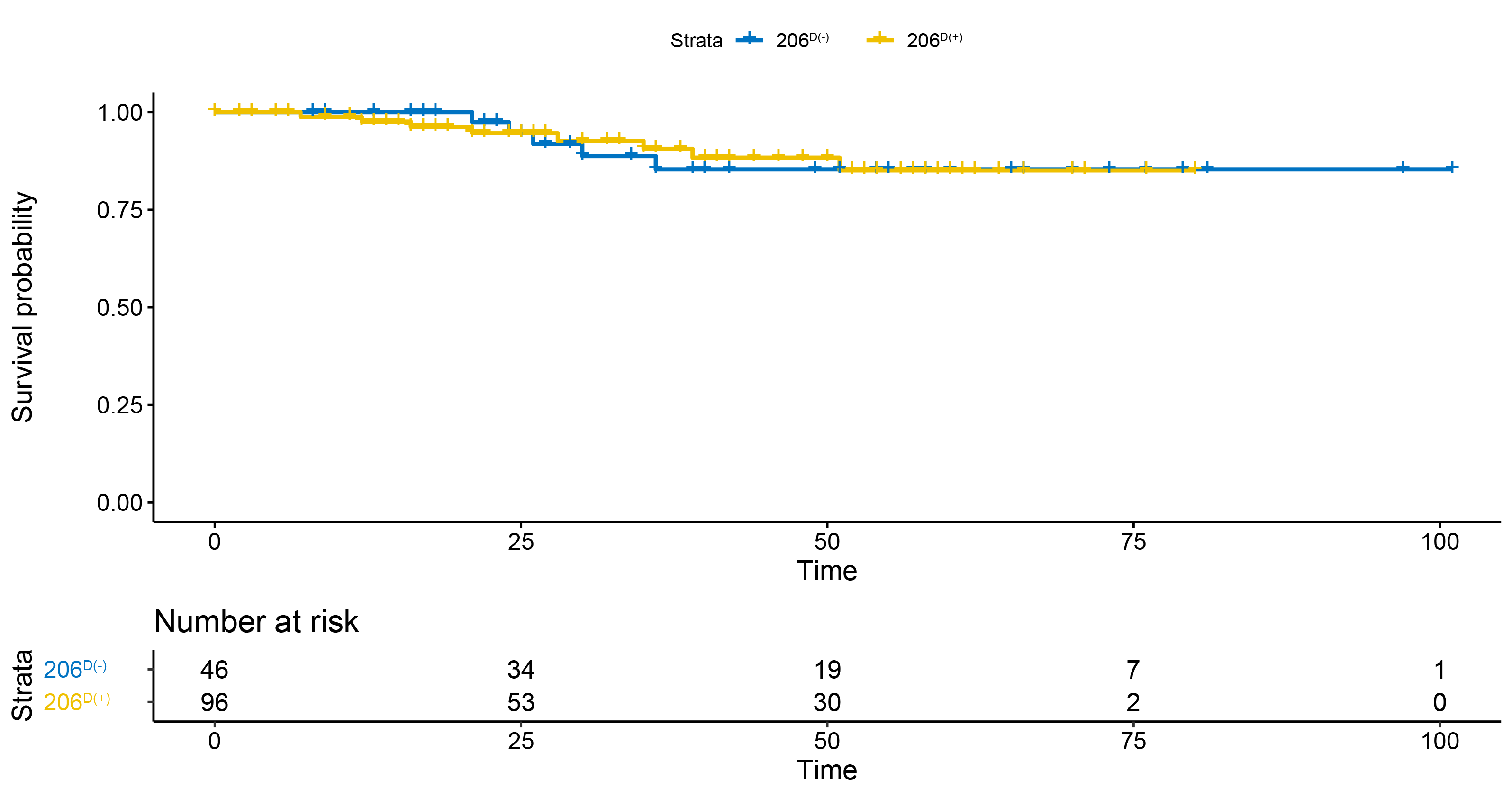

Supplement: Supplementary file 5 — Figure S2 [file CAM4-11-2366-s002.zip › figure/CAM4_4626_Supplementary_Figure2_b.tif]

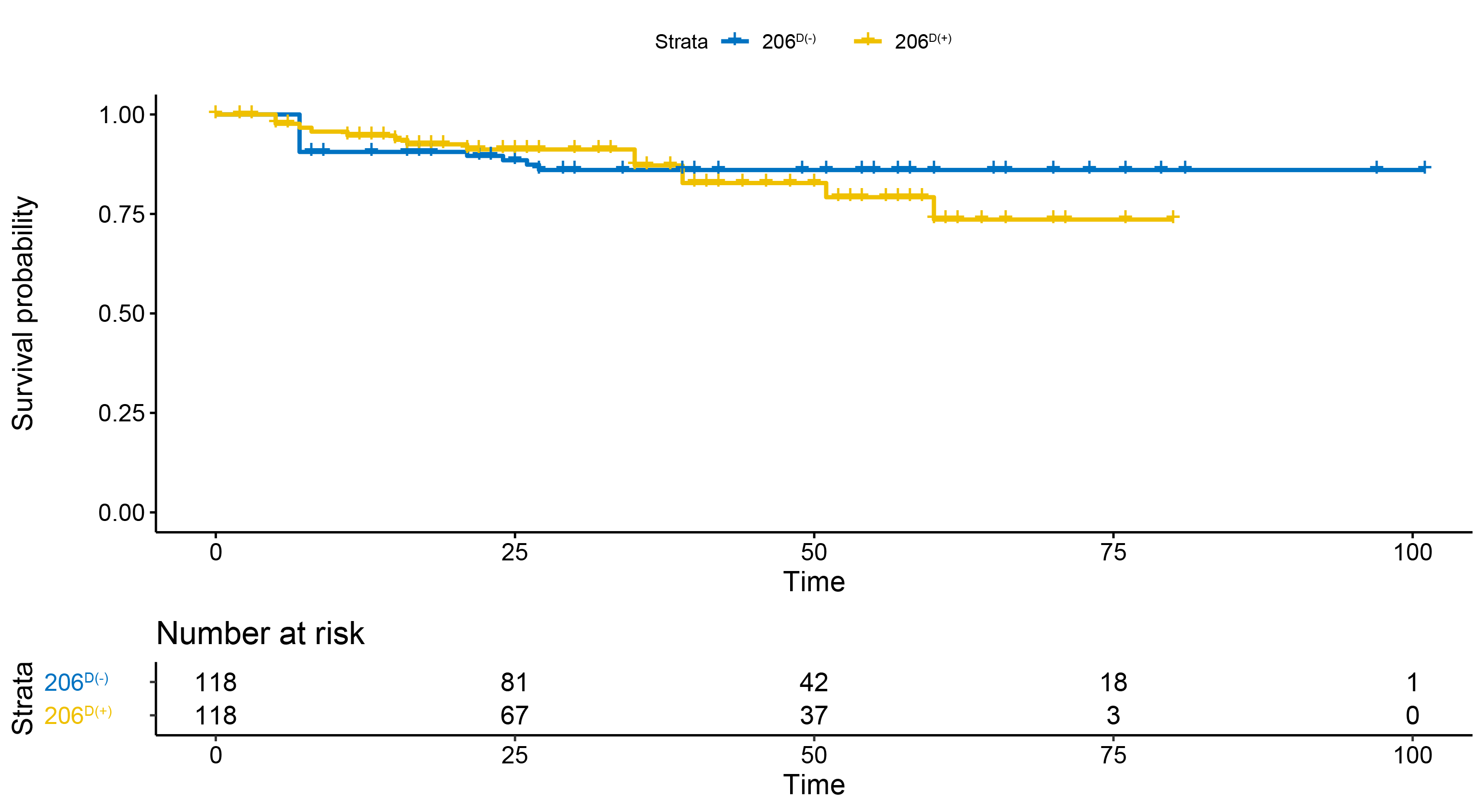

Supplement: Supplementary file 5 — Figure S2 [file CAM4-11-2366-s002.zip › figure/CAM4_4626_Supplementary_Figure2_c.tif]

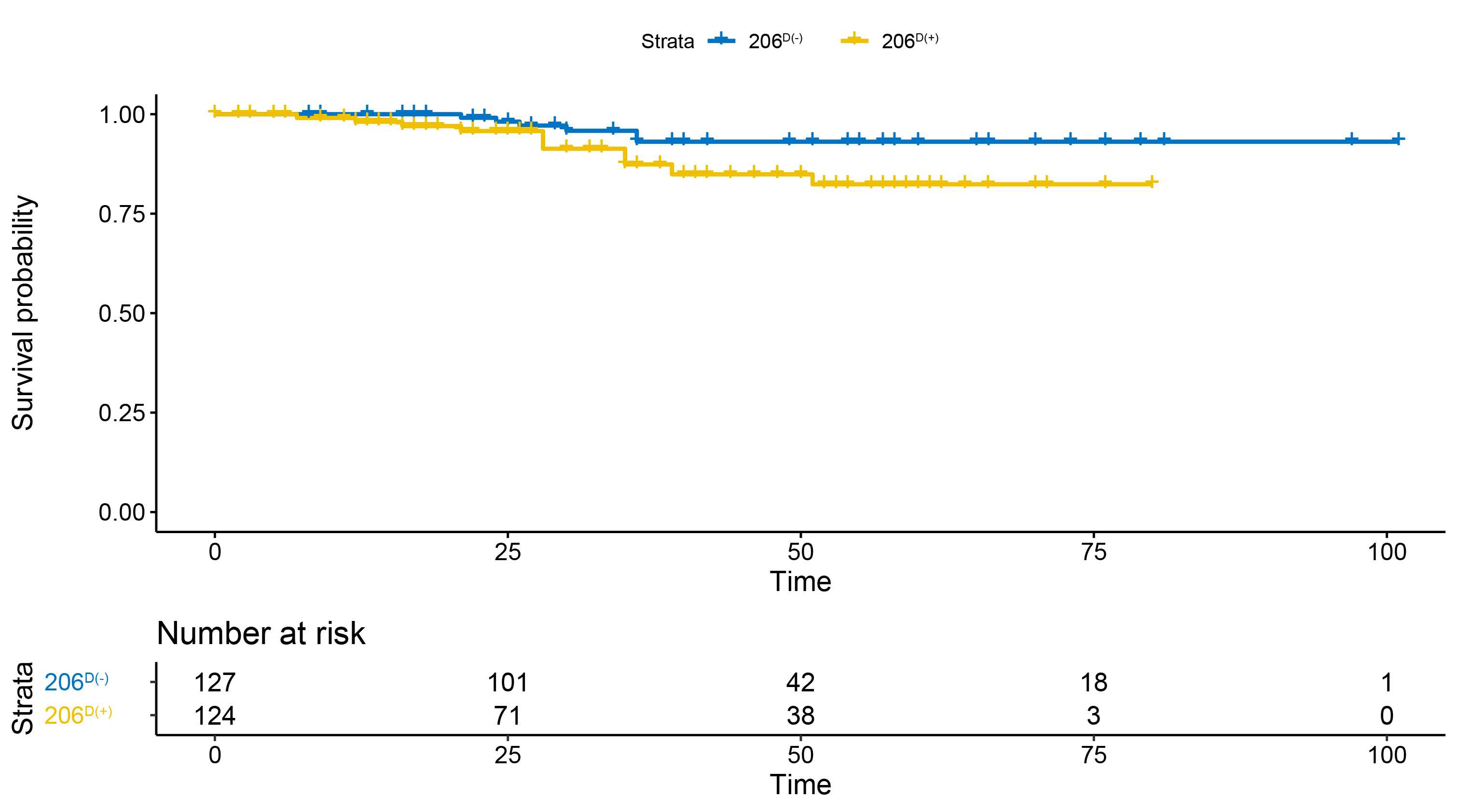

Supplement: Supplementary file 5 — Figure S2 [file CAM4-11-2366-s002.zip › figure/CAM4_4626_Supplementary_Figure2_d.tif]

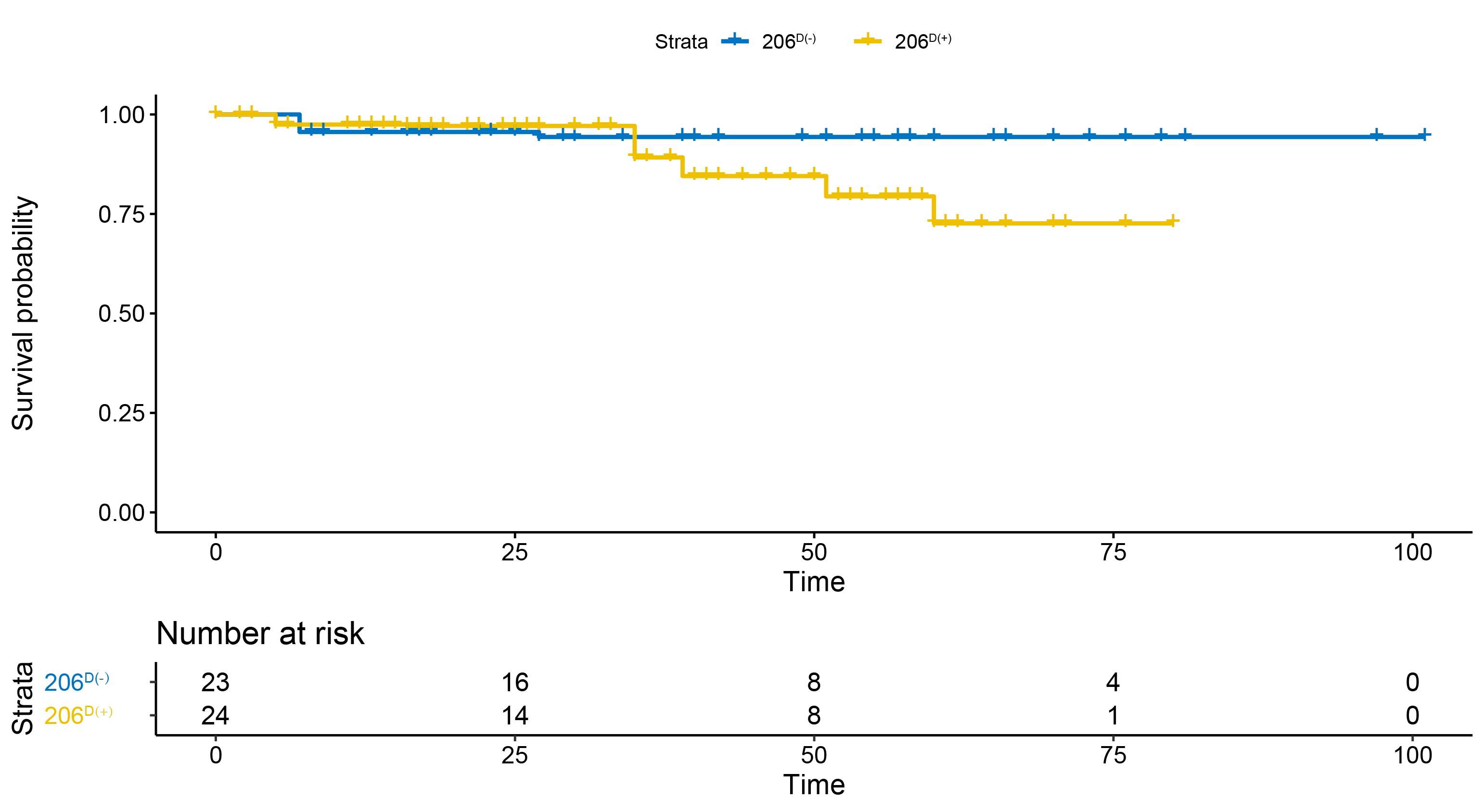

Supplement: Supplementary file 5 — Figure S2 [file CAM4-11-2366-s002.zip › figure/CAM4_4626_Supplementary_Figure2_e.tif]

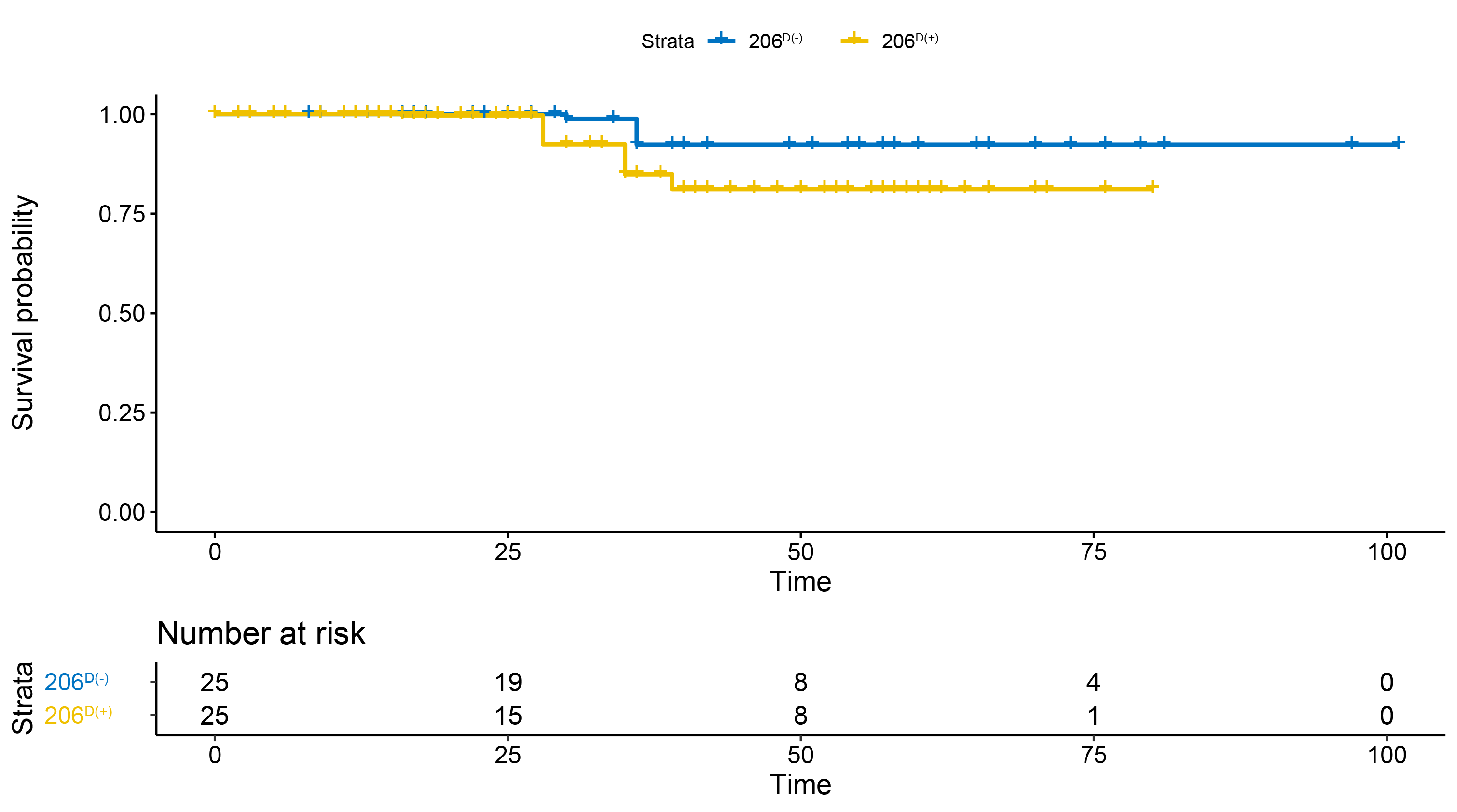

Supplement: Supplementary file 5 — Figure S2 [file CAM4-11-2366-s002.zip › figure/CAM4_4626_Supplementary_Figure2_f.tif]
